# Processes supporting effective skill-mix implementation in general practice: A qualitative study

**DOI:** 10.1177/13558196221091356

**Published:** 2022-05-03

**Authors:** Sharon Spooner, Imelda McDermott, Mhorag Goff, Damian Hodgson, Anne McBride, Katherine Checkland

**Affiliations:** 1 Centre for Primary Care Research, 12203 University of Manchester, UK; 2 Management School, 7315 University of Sheffield, UK; 3 Alliance Manchester Business School, 12203 University of Manchester, UK

**Keywords:** workforce, multidisciplinary, qualitative

## Abstract

**Objectives:**

Health policy and funding initiatives have addressed increasing workloads in general practice through the deployment of clinicians from different disciplinary backgrounds. This study examines how general practices in England operate with increasingly diverse groups of practitioners.

**Methods:**

Five general practices were selected for maximum variation of the duration and diversity of skill-mix in their workforce. Individual interviews were recorded with management and administrative staff and different types of practitioner. Patient surveys and focus groups gathered patients’ perspectives of consulting with different practitioners. Researchers collaborated during coding and thematic analysis of transcripts of audio recordings.

**Results:**

The introduction of a wide range of practitioners required significant changes in how practices dealt with patients requesting treatment, and these changes were not necessarily straightforward. The matching of patients with practitioners required effective categorization of health care patients’ reported problem(s) and an understanding of practitioners’ capabilities. We identified individual and organizational responses that could minimize the impact on patients, practitioners and practices of imperfections in the matching process.

**Conclusions:**

The processes underpinning the redistribution of tasks from GPs to non-GP practitioners are complex. As practitioner employment under the Primary Care Network contracts continues to increase, it is not clear how the necessarily fine-grained adjustments will be made for practitioners working across multiple practices.

## Introduction

There is a workforce crisis in UK primary care, with general practitioner (GP) numbers falling as demand for care increases.^1,2^ Government health policy has sought to address workload pressures through diversification of the primary care workforce, often described as a change in ‘skill mix’.^3(p7)^ Skill-mix changes are intended to reduce pressure on GP appointments on the premise that, through organizational processes such as delegation or substitution, some work traditionally done by GPs can safely and effectively be transferred to non-GP practitioners.^4,5^ The most recent manifestation of this policy is the subsidized employment of a wide range of practitioners across networks of practices known as Primary Care Networks.^6^

An additional implicit assumption underlying moves towards skill-mix change is that work can be divided into discrete tasks and allocated to workers equipped with the capacity necessary to undertake them. Indeed, international studies of task redistribution in hospital settings and of costs associated with skill-mix implementation indicate that improved health care delivery at lower cost is possible.^7,8^ However, whilst research studies have described the contribution made by different types of practitioner in general practice settings,^9-11^ there is limited evidence about how best to distribute or perform the broad spectrum of unfiltered, undifferentiated work that patients bring to general practice.^12,13^ Furthermore, studies report ‘ambiguity on the purpose and place of new roles’*,* together with variation in how new roles are assimilated and in how practitioners expect to work.^14(e496)^ Lessons from the wider literature on organizational change in primary care indicate that practices are complex, and that change processes can trigger a complex set of emergent changes and adaptations throughout different layers of the organization.^15^ This suggests that, rather than conceptualizing skill-mix change as a straightforward implementation task, it can best be considered as a significant and evolving change in a complex system that will require and generate widespread and not necessarily predictable adaptations to organizational processes and routines.^16^

As funding support for skill-mix implementation through Primary Care Networks increases, detailed evidence is urgently needed about how these changes play out and how working practices need to be adjusted to optimize the benefits of skill-mix.^12,17^ This study aims to capture and explore the adaptations that occur as practices accommodate new practitioners and new ways of working.

As independent contractors, general practices deliver services according to their contracts whilst holding responsibility for staff employment and management.^6^ Growing numbers of advanced nurse and clinical practitioners, physician associates, clinical pharmacists, paramedics and physiotherapists are now employed alongside GPs and practice nurses.^18^

Safely and effectively distributing varied work across a group of practitioners with differing skills and experience relies on allocating patients/problems to practitioners capable of dealing with them. However, since general practice deals with unfiltered, undifferentiated caseloads, practices need processes that ensure that the right patient, is seen by the right practitioner, with the right training, in the right place, at the right time.^19^ Research suggests that realization of the benefits of health care workforce changes is contingent on avoiding duplication, fragmentation, increased costs or loss of patient confidence.^20^

Recognizing the potential impact of complex effects that may accompany changes in workforce composition within organizations, this paper draws on our analysis of a detailed case study across five general practices, addressing the research questions:

• How do practices accommodate skill-mix change in their daily work?
• How do practitioners, practice staff and patients experience these processes?

## Methods

We undertook qualitative case studies in five general practices. Our approach was broadly informed by the interpretivist tradition,^21^ with interview responses considered as an expression of underlying meanings as well as imparting information. Our use of both observation and interviews allowed exploration of discrepancies between work-as-described and work-as-done, deepening our analysis. Fieldwork was conducted during August–December 2019, prior to COVID-19.

Practices were selected to include those with diverse workforces including, for example physician associates, advanced clinical practitioners and clinical pharmacists in patient-facing roles. We recruited practices which had had a more mixed workforce for some time, as we were interested in understanding the processes of adjustment over time, but we also recruited a late-adopter practice to allow comparison and to capture early experiences. Table 1 sets out site characteristics.

**Table 1.** Site and practitioner workforce characteristics.

Site	A	B	C	D	E
Location	Small town, semi-rural	Small town, semi-rural	City (multi-site practice)	Town and rural surrounds	City
Maturity of skill-mix^a^	Early adopter	Late adopter	Early adopter	Early adopter	Early adopter
Registered patients (approx.)	11,000	14,000	59,000	17,000	10,000
Workforce					
Advanced clinical practitioner	2	2	0	0	0
Advanced nurse practitioner	0	0	2	4	1
Clinical pharmacist	1	1	5	0	1
GP partners	7	6	>20	8	3
GP salaried	0	3		4	5
Physician associate	0	0	1	0	2
Practice nurse	0	4	0	2	2
Others	Advanced clinical practitioner trainees (1 paramedic 1 nurse), GP registrars, Health care assistants, Health visitors, Midwife	Community midwife, Health care assistants	First contact physiotherapist, Health care assistants, Phlebotomist, Social prescriber, Specialist nurses, Urgent care practitioners (1 training as advanced clinical practitioner)	Health care assistants, Medicine management team, Nurse lead, Phlebotomists, Research nurse, Treatment room nurses	Community nurses, Health care assistants, Health visitors

^a^The term ‘early adopter’ refers to practices that adopted skill-mix in 2016 or earlier and the ‘late adopter’ adopted skill-mix in 2018.

Three researchers spent approximately 6 weeks in each practice, with each researcher taking overall responsibility for one or more sites to allow the development of trusting relationships, although researchers visited other sites to support fieldwork. All researchers are experienced in qualitative research, one (SS) is also a GP. After familiarization and an introductory interview with the practice manager, researchers spent time with clinical practitioners in each site, observing consultations and engaging in informal discussions. Formal semi-structured interviews were carried out to explore their perceptions of their roles and of the factors supporting or inhibiting their work. Staff meetings were observed, alongside observation in informal settings such as coffee rooms. Researchers engaged in informal conversations with observed practitioners and were therefore able to conduct near-real-time sense-checking of their understanding of observed behaviours.

We also observed in reception areas and telephone rooms to understand how patients were allocated to practitioners. Receptionists were interviewed to capture their perceptions. Informed consent was sought and patients were informed about the research via posters in reception areas. Patients arriving for an observed session were provided with an information sheet and asked for consent to the presence of a researcher. Detailed field notes were kept capturing organizational processes, the nature of clinician-patient interactions and the extent to which practitioners liaised with colleagues or sought support during or between consultations. Initial topic guides for the semi-structured interviews were derived from preliminary review of the literature, and adapted to take account of findings from observations. Table 2 sets out the data collected.

**Table 2.** Numbers of interview (Int) and observation (Obs) participants.

Roles	Sites											TOTALS
	A		B		C		D		E			
	Int	Obs	Int	Obs	Int	Obs	Int	Obs	Int	Obs	Int	Obs
Advanced clinical practitioner (including trainees)	1	0	1	1	2	2	0	0	0	0	*4*	*3*
Advanced nurse practitioner	1	2	0	0	1	1	1	2	1	0	*4*	*5*
Clinical pharmacist	1	0	1	1	1	0	1	0	1	1	*5*	*2*
GP	2	2	2	2	2	1	2	1	1	1	*9*	*7*
Paramedic	1	1	0	0	0	0	0	0	0	0	*1*	*1*
Physician associate	0	0	0	0	1	1	0	0	2	2	*3*	*3*
Practice manager	1	0	1	0	0	0	2	0	1	0	*5*	*0*
Practice nurse	1	2	0	0	0	0	0	0	0	0	*1*	*2*
Receptionist	1	1	1	0	1	1	1	1	1	1	*5*	*4*
Social prescriber	0	0	0	0	1	0	0	0	0	0	*1*	*0*
*TOTAL*	*9*	*8*	*6*	*4*	*9*	*6*	*7*	*4*	*7*	*5*	*38*	*27*

To understand patient perspectives on skill-mix change we undertook patient surveys in each practice. A short survey was developed with the help of representatives from a public and patient forum who were supporting the research, and distributed to patients attending the practice during site visits. A total of 125 surveys were obtained over the five sites. In addition we carried out focus groups with members of the practice Patient Participation Group (i.e. a group of patients, carers and GP practice staff, who meet to discuss practice issues and patient experience) in four out of the five sites (the fifth site had no active group) and one group of patients who were not linked with the Patient Participation Group. These discussions explored patients’ perceptions of skill-mix in their practices, and participants were invited to reflect upon what worked well and what they felt could be done better.

We developed an initial coding framework based around our understanding of the relevant literature and theory and supplemented with additional codes during continuing iterative analysis. We developed narrative case descriptions, synthesizing observational and interview data to describe how and why skill-mix change had occurred and how it was managed. In keeping with our overall research questions, we particularly focused upon understanding patient journeys. Having recognized that skill-mix change is a complex set of processes rather than a standardized intervention our analysis drew on Stake’^22^s approach to holistically and interpretively analyse data across all study sites. As we analysed how each practice operated, this allowed us to maintain focus on phenomena within bounded but distinct operational systems within the overall case. Thematic coding of interviews was informed by field notes, and memos generated during analysis helped us capture theoretical ideas and develop second order analysis which we refined following discussion at team meetings. This supported theoretical generalization from our data to provide a broader understanding of the processes reported and observed during implementation of increased skill-mix diversity in general practice.

Our analysis across all cases highlighted the importance of categorization and matching processes not previously described in the literature, and these were added to the code list. Examination of early and late adopting practices also highlighted the dynamic nature of the processes required to accommodate skill-mix change and the need for flexibility and adaptability over time. Patient views and experiences were initially coded separately, but then integrated into the overall analysis in team discussions as we explored how the processes we were describing were experienced by patients as well as staff.

## Results

### Categorization of patients’ problems

Patients request appointments in general practices to talk about relatively undifferentiated problems, which may range from urgent and life-threatening conditions to a wide variety of ill-defined, chronic, long term and complex problems. Whilst GPs can typically deal with every different type of problem, the new types of practitioners entering general practice (such as physician associates and advanced clinical practitioners) have different training, different skills and hence different scopes of practice. To ensure that patients see a practitioner who can deal with their problems it is first necessary to attempt to categorize those problems.

Categorization is defined as ‘the process of dividing the world into groups of entities whose members are in some way similar to each other’.^23(p518)^ In our case study sites this was done verbally or using an online or AI-enabled platform. Details were typically received by a non-clinical receptionist with training in asking about symptoms and general health issues. Whilst key ‘red flag’ conditions were readily recognized as urgent or life-threatening and dealt with according to locally agreed protocols,^24^ other problems were more nuanced.

Interviews with GP practice staff and patients indicated that this categorization process could sometimes be problematic. Firstly, patients did not always feel comfortable about disclosing details to non-clinical staff, whether because of concerns about confidentiality or lack of confidence in a receptionist’s ability to understand and provide the best appointment. Practices attempted to allay such concerns using phone-answering messages:

I can’t remember the exact words but along the lines of, ‘In order that you get to see the right clinician for the right amount of time the receptionist will need to ask you some questions.’ (Site B, practice manager)

Whilst acknowledging that some patients would refuse, for example ‘I’m not telling some lowly receptionist anything’ (Site B, practice manager), practice staff sought to gradually shift patients’ attitudes, ensuring that receptionists could seek advice from experienced clinicians when necessary. At one site, patients seemed to find submitting information via an online form more acceptable than direct communication. However, patients perceived that they had little choice about which practitioner dealt with their problems since ‘It’s just the receptionist that decides’ (Site D, patient focus group).

Part of the difficulty with categorization lay in the unfiltered, undifferentiated nature of problems presented by patients and the limitless and sometimes confused ways in which problems are articulated. Managers recognized that it was infeasible to train reception staff to make clinical decisions and on occasion the reported problem did not match the problem as eventually presented:

Staff are not as skilled. They’re not trained enough to make clinical decisions. And neither should they be, that’s not safe either. (Site B, practice manager)

They’ll write down why [the patients are] coming in, but it may be completely...different. (Site E, advanced nurse practitioner).

### Categorization of practitioners’ skills

Standardized descriptions of the training programmes, qualifications, skill sets, or competencies of non-medical practitioners employed in UK general practice have not yet been fully developed. Guidance in the form of a ‘route to practice’ is emerging for some types of practitioners, but for many types of practitioner no singular pathway to practice has been set out, and this in itself contributes to a lack of clarity about what each practitioner can do.

In addition to categorizing the problem, then, practice staff categorized practitioners’ skills*.* This was particularly important in practices with the most diverse clinical workforce.

To some extent, receptionists allocated work according to the roles specific practitioners were employed to do:

We have nurses that specialise in vascular and COPD, we have diabetes nurses, we have Prescribing Team nurses as well, and we also have nurses that work specifically in those teams, but don’t prescribe, so we have an Asthma Team, Vascular Team, Dementia Team, COPD, Diabetes, Hypertension. (Site D, receptionist)

However, whilst receptionists spoke about [information] ‘sheets in reception of ACP [advanced clinical practitioner] and ANP [advanced nurse practitioner] capabilities’ (Site A, receptionist), the process became more complex when practitioners occupying the same role functioned differently from each other, as illustrated by two advanced nurse practitioners’ caseload descriptions:

My job is basically to see patients on undifferentiated presentation, and I will see everything that a doctor will see. Without exception. (Site C, advanced nurse practitioner)

[I] don’t see pregnant women… I don’t do pathology. I don’t do the blood test results. I don’t often see, you know, investigations. The GPs will often take ownership of the investigations…for example, you know, an ultrasound scan or a chest x-ray...the results will go to the GP… I mean, obviously, they do all the higher end work. (Site E, advanced nurse practitioner)

In practice, the competencies of role holders were dependent on factors such as additional qualifications, clinical experience, and individual strengths and limitations. In the absence of predefined role competencies, practices developed their own competency frameworks, recording information about individual practitioners’ competencies in what numerous participants termed a ‘skills matrix’ or ‘bible’ that receptionists referred to. These were annotated or updated as practitioners gained additional competencies or changed role:

We’ve had to start in primary care and invent our own competency frameworks and sort of ways of working. (Site C, GP)

We have to make sure we know what people are doing, so that we’ve got the most up-to-date information and so that we are putting people in with the right clinicians. (Site A, receptionist)

Updating the skills matrix revealed that practitioners from non-medical backgrounds with limited training or clinical experience were restricted in what they could contribute:

[Physician associates] were nice, but we were very restricted on what we could put with them, on the skills matrix...because they come in mainly from a non-medical background, and just do like a year’s intensive course, or is it two years’?, I can’t remember. (Site D, receptionist)

Such skills matrices were both practice and practitioner-specific, and required frequent updating.

### Assigning work to a practitioner able to deal with the problem

Matching work with the ‘right’ practitioner could simply be a matter of recognizing that the request fitted predefined patterns of work distribution:

If it’s a medications review it goes to [a clinical pharmacist] and if it’s a frail and elderly type person it will go to [the advanced clinical practitioners]. (Site B, practice manager)

However, achieving a good match through categorization of the problem and practitioner can be difficult, particularly when appointment availability is restricted:

There’s always going to be the odd error in the system but that’s where you look and you think, well, I can’t prescribe something for that infected toe, it needs to be seen by someone else. (Site A, practice nurse)

With the pressure of appointments and, you know, the demand of the patient, you know, just sometimes the receptionist will book an inappropriate appointment. And we learn, you know. So where that came from was the nurses’ meeting last week...where the reception manager was in there and the nurses were saying, well, you know, this appointment was made, and it wasn’t right, you know, so we learn. (Site A, practice manager)

Practitioners with generalist skills are particularly useful to deal with the very wide variety of problems and a high prevalence of co-existing conditions:

I think there’s a real room for generalists like me a little bit...you want people to have the general practice nurse...general primary care skill mix and not be too specialised, because, of course, patients walk in with all of it. (Site E, advanced nurse practitioner)

However, since such practitioners are in limited supply, practices improve overall access by delegating specific categories of work. For example some clinical pharmacists focus on medication reviews and audits, whilst additional training and experience allows others to undertake NHS health checks, disease monitoring, treatments for minor ailments, and prescribing.

In addition, practices sought to increase options for matching by supporting practitioners to upgrade their skills and work more independently:

We’ve also encouraged that kind of middle tier of nurses to become independent prescribers, so they can manage things right the way through without the need for knocking on somebody’s door to get prescriptions out. (Site D, GP)

### Flexible strategies that improve experiences and outcomes for patients and practitioners

Given the complexities of categorization and matching processes, imperfect categorization or mismatching is inevitable, leading to practitioners facing problems outside their usual scope of practice. Observed examples of mismatching included staff unable to administer required injections, staff unable to authorize prescriptions or certification, staff unable to alter medication, staff having problems because the required skills were outside practitioner’s skillset (e.g. mental health issues), and staff unable to deal with multiple problems in one consultation. Organizational responses to such mismatching incidents varied from an informal note or reminder to reception staff, to staff meetings that led to changes in the processes for distributing work.

We identified three levels at which flexibility was used to improve the categorization-matching process.

### Organizational level

We observed that adjustments had been made to the work schedules of senior clinicians to facilitate their direct involvement in triage and allow them to promptly provide clinical support or advice:

[The practice has] a system where one doctor per day has triage duties. They may have other duties as well. (Site A, patient focus group)

[Non-GP practitioners] can consult either by getting a doctor into the consulting room within minutes, or agreeing to discuss it with the doctor that day and then you get a call back as to whether it’s considered necessary to make an appointment with the doctor or not (Site A, patient focus group)

Similarly, a team approach to triage of appointment requests increased opportunities for diverse practitioners to learn more from and about each other:

GPs work closely to understand what [advanced nurse practitioners] can do. They’ll meet more regularly to discuss patients. And that’s been invaluable for us...The understanding and communication they have between them now is far better since we implemented that team, because they’re all on that team on a regular basis. (Site D, practice manager)

### Practitioner level

Practitioners sometimes tried to ensure that more complex patients were allocated additional time:

I’ve tried with the reception staff for patients who are on more than eight or 10 items of medication to do a double appointment. Some of them are very good at doing that, others aren’t. (Site B, clinical pharmacist)

Having insufficient time to undertake a proper review could prolong the pharmacist’s working day:

I’ve had to set up a laptop that I take home with me, so that I can see the kids and then I can log on and I can do all my clinical work. (Site C, GP)

On other occasions, an ‘escalation’ response was required when the problem was beyond a practitioner’s capabilities. Such flexibility was facilitated at Site A by having a GP rostered to cover triage inquiries and escalation cases rather than being fully booked with their own consultations:

We tend to have a triage GP every day, so I could possibly say, ‘I’ve got this booked in with me, I don’t think it’s appropriate…could you come and have a look at it.’ (Site A, practice nurse)

Patients reported that such rapid resolution supported their needs:

I have personal experience of coming to see a nurse, which I was very happy with, on a relatively mundane issue, which was to do with one of my ears. And immediately there was escalation. She…got one of the doctors, a senior doctor, who came and looked within minutes. (Site A, patient focus group)

When they were unable to deal with all the problems presented by patient, practitioners opted for a selective approach, making progress on what seemed important (and which the patient might mention early in the consultation) and deferring action on less urgent issues (mentioned as second, third or fourth problems) for another occasion:

Even if I’m not solving the problem I can do the groundwork sometimes for the GPs, in terms of taking the history, organising the bloods…Now, I’m more than happy to go the extra mile for my patients, but not when I’m running an hour late, and you’ve had this problem for months, so it doesn’t actually necessarily, need to be done today. (Site E, advanced nurse practitioner)

I know it is difficult to get appointments and we are supposed to say, ‘Go away and come back another day.’ But I think we’re all very nice here which sometimes gets us into trouble and makes us run late, and it’s finding that balance. I think what I normally do is I’d triage the [patient’s] second, third and fourth problems to see how serious [they really are]. (Site E, physician associate)

### Patient level

Practices recognized that changes in how appointments were allocated affected patients. Interviews with both patients and practitioners revealed that many patients remained uncertain about what some practitioners could do:

Some of them understand it. Some of them are a bit like ‘You could do this’ and I was like, ‘No, I can’t do that’…‘Can you change this medication for me, my depression?’ I was like ‘I can’t do that. You do need to see a doctor for that.’ (Site A, practice nurse)

Many patients were unconcerned about what type of practitioner they saw, just so long as their needs were met. They particularly valued reassurance that flexible mechanisms were available to practitioners dealing with anything beyond their capabilities:

Fundamentally, as long as people think that you are able to manage their problem, they’re not too interested in the difference [in practitioners]…People aren’t really bothered as long as you can manage what they need, and nine times out of 10 that’s not an issue. (Site E, physician associate)

[Physician associates generally] don’t just bumble along doing things…but if they’re not happy they’ll put their hand up and say, ‘I’m out of my depth, you need to go and see a doctor.’ (Site E, patient focus group)

Over time, and with experience of seeing different practitioners, some patients grew to prefer consultations with non-GP practitioners. In part, this was because patients felt non-GP practitioners looked at their issues more thoroughly, as they would usually have a longer, more holistic, consultation:

[Nurse practitioners] are senior nurses not GPs, I think they take more, not ‘care’, because that sounds like I don’t trust the doctors, and I don’t mean that, but I think it’s a different mindset, I think they’re more thorough. (Site D, patient focus group)

The senior nurse is very, very thorough, and so is the physician associate…In fact, I prefer to go and see them rather than go and see the GP. (Site E, patient focus group)

Rather than feel worried when a problem was escalated to a colleague, patients expressed ‘an enormous sense of relief’ (Site E, patient focus group) and increased confidence that further help was available. Indeed, patients were concerned if practitioners continued with what they perceived were ineffective treatments:

I had some weird experiences with nurses that just keep prescribing antibiotics and not looking at the underlying symptoms. (Site D, patient focus group)

## Discussion

Studies of skill-mix implementation in non-GP settings has shown that transferring protocol-driven tasks from doctors to nurses is safe for patients and that substituting nurses for doctors can achieve broadly similar outcomes.^26-28^ But to our knowledge, no previous studies have examined the practical processes necessary to accommodate skill-mix change in primary care settings. What studies do exist have tended to focus on the tasks undertaken by practitioners rather than report clearly on the processes by which tasks were distributed across clinical teams.^29^

This paper shifts the focus from seeking a theoretical but undefined ideal skill-mix workforce composition to considering how practices can optimize their performance by improving how work is distributed across practice teams. As primary care is increasingly provided by practitioners with diverse skills and experience, it is important that work is safely and effectively distributed. Our results indicate that three key components underpin this:

• *Categorisation* of each patient’s problem and each practitioner’s skillset
• *Matching* the problem, skillset and availability of appointments
• *Flexibility* in making any necessary, timely adjustments to the initial matching result.

Whilst such processes have been part of general practice since the introduction of practice nurses, we found that the increasing diversity of the practitioner workforce requires more complex and adaptable organizational processes. Given the wide range of undifferentiated problems presenting in primary care it is inevitable that patients will sometimes see a practitioner who cannot deal with their problem. This brings inefficiencies for practices and patients alike. However if workplace organization enables sufficient flexibility, practitioners can more easily address all aspects of care in a timely manner.

Our findings raise significant issues for the current roll out of skill-mix change in England via Primary Care Networks. Under the new Network contract (an add-on to the General Medical Services Contract) subsidized practitioners such as clinical pharmacists, physician associates and advanced practitioners will work across a number of practices in a network. This creates additional difficulty with the detailed work of categorizing and matching. Where these processes worked in our study, they did so because managers, administrative and clinical staff working closely together were able to distribute work and work flexibly through knowing each other’s capabilities and limitations. Adaptation over time was particularly important. How this will work when practitioners move between practices, only spending short periods of time in each practice, is not clear.

Moreover, the flexible processes that we found facilitated skill-mix implementation were practice and context-specific. This suggests that new practitioners employed across Primary Care Networks will need to adapt flexibly to different working environments, potentially working differently in different practices. For instance, our study found that skills matrices were both practice and practitioner-specific, and required frequent updating. It seems likely that updating skills matrices will become increasingly difficult when practitioners are employed across multiple practices, as is envisaged in Primary Care Networks. Our data gathering ended before any impact of this could be observed, suggesting this issue requires further research.

### Limitations

This study has two main limitations. Firstly, our intention to explore in detail whether operational maturity might be more evident in the processes adopted by practices with longer experience of skill-mix (i.e. early adopters) than in practices where skill-mix was more recently introduced, was unachievable due to the difficulty we experienced recruiting sites fitting the latter description. Whilst additional strategies to improve how practices undertake each part of the process may emerge from a larger-scale study, the general principles we identified can be applied in most practice settings to support the implementation of skill-mix.

Secondly, this study reveals only part of a lengthier, possibly more fraught, process for patients and practices when skill-mix increases. It seems likely that an additional consequence of increasing skill-mix in general practice may be a reduction in continuity of care. This may be a concern, as greater continuity of care has been shown to lead to better patient outcomes.^25^ A detailed discussion of outcomes associated with skill-mix changes lies outside the scope of this process-focused paper.^30^

## Conclusions

Our research suggests that any search for an ‘optimal’ skill-mix is likely to be futile.^31^ That is because of the undifferentiated nature of problems presenting in general practice and the lack of standardization of skills and capabilities between practitioners. Rather, our exploration into how practices accommodate skill-mix change in their daily work and how practitioners, practice staff and patients experience these changes suggests that to successfully adapt to skill-mix change, practice staff and patients must negotiate additional layers of complexity in how health problems are presented for categorization, how work is distributed to match the capabilities of practitioners and how any mismatching is managed to minimize detrimental impact.
